# Shared diagnostic biomarkers in metabolic syndrome and coronary artery disease identified by integrated bioinformatics and machine learning

**DOI:** 10.1530/EC-26-0240

**Published:** 2026-06-08

**Authors:** Nuojin Wang, Keyan Hu, Shuting Zhang, Hongxing Zhao, Tianrong Pan, Tong Wang, Yi Zhang, Xueying Liu

**Affiliations:** ^1^ Department of Endocrinology, The Second Affiliated Hospital of Anhui Medical University, Hefei, Anhui, China; ^2^ Department of Endocrinology, The First Affiliated Hospital, and College of Clinical Medicine of Henan University of Science and Technology, Luoyang, Henan, China; ^3^ General Practice, The Second Affiliated Hospital of Anhui Medical University, Hefei, Anhui, China; ^4^ Department of Endocrinology, The First Affiliated Hospital of Anhui Medical University, Hefei, Anhui, China

**Keywords:** metabolic syndrome, coronary artery disease, bioinformatics, diagnostic biomarkers, hub genes

## Abstract

**Background:**

Metabolic syndrome (MetS) comprises various complicated metabolic disorders. Coronary artery disease (CAD) is a major cardiovascular disease worldwide. These two diseases are the principal causes of morbidity and mortality in older adults. Previous studies have suggested a potential link between these two diseases. To date, finding sensitive and effective diagnostic biomarkers of MetS and CAD remains challenging. This research examined the potential shared biomarkers of MetS and CAD using a comprehensive bioinformatics approach.

**Methods:**

Five microarray datasets about MetS and CAD were used in our analysis. Integrated bioinformatics methods, such as differentially expressed gene (DEG) analysis, weighted gene co-expression network analysis (WGCNA), and machine learning (ML) algorithms, were utilized to discern hub genes of MetS and CAD. Meanwhile, single-sample gene enrichment analysis and single-cell analysis were leveraged to analyze immune cell infiltration and the abundance of gene expression in immune cells. Finally, the expression levels of key biomarkers were primarily verified by RT-qPCR.

**Results:**

We identified 666 DEGs associated with MetS and 762 DEGs related to CAD, with 22 overlapping genes. Meanwhile, 3 hub genes related to MetS and CAD were screened by WGCNA, which were *APOBEC3B, SGSM2,* and *LRRC32*. Two hub genes, *ADRB2* and *KDM6A*, were downregulated in the two diseases. ROC curves confirmed their diagnostic value. Furthermore, single-cell sequencing analysis and RT-qPCR obtained consistent findings.

**Conclusion:**

*ADRB2* and *KDM6A* are identified as hub candidate diagnostic biomarkers for MetS and CAD. These genes may offer new targets for MetS and CAD and assist in exploring the molecular mechanisms underlying both diseases.

## Introduction

Coronary artery disease (CAD) is a prevailing cardiovascular disease (CVD) resulting from atherosclerosis, affecting millions of individuals worldwide. It is devastating because patients, especially young people, may die or become disabled without signs (1). In a nationwide PCI registry of STEMI patients, individuals aged <40 years account for 3–4% of cases (2). Among asymptomatic adults aged 40–54 years with subclinical atherosclerosis, approximately 63% of participants are found to have plaque or coronary artery calcification defined by imaging. Such a substantial proportion of CAD can cause high rates of morbidity and mortality.

Metabolic syndrome (MetS) is an intricate disorder marked by three or more of the following conditions: central obesity, hyperglycemia and/or diabetes mellitus (DM), hypertension, increased triglyceride levels, and reduced high-density lipoprotein cholesterol levels (3). The prevalence of MetS varies substantially across ethnic and demographic groups and depends on diagnostic criteria. In high-income countries, the overall prevalence of MetS has increased from 24.2 to 31.9% over recent decades (4, 5). MetS and its components are closely linked with the overall mortality of CVDs, making it a serious health concern and economic burden (5, 6).

MetS is tightly linked with obesity, adipose tissue dysfunction, endothelial dysfunction, and vascular stiffness, which are involved in the pathology and physiology of DM and CAD (7). As the prevalence of obesity, DM, hypertension, and dyslipidemia has risen sharply worldwide (8, 9, 10, 11), MetS has become a fundamental public health concern, especially in the primary prevention and early diagnosis of CAD. However, early detection of MetS and CAD is challenging due to the paucity of specific biomarkers. Better methods are pressingly needed to distinguish such populations based on available clinical variables.

Bioinformatics tools integrate and analyze data to predict potential biomarkers and the molecular pathogenesis of diseases more accurately, thus accelerating the development of precision medicine. This study investigated the specific biomarkers and underlying pathways in MetS and CAD. Differentially expressed genes (DEGs) and critical modules of MetS and CAD were acquired from public databases. Thereby, three machine learning (ML) algorithms and ROC curves were leveraged to discern the shared diagnostic genes and confirm their diagnostic value for MetS and CAD. The role of immune cells in MetS and CAD was also examined through single-sample gene set enrichment analysis (ssGSEA). This paper offers valuable ideas for the shared mechanisms of MetS and CAD and underlines the potential of β2-adrenergic receptor (*ADRB2*) and lysine demethylase 6A (*KDM6A*) as candidate diagnostic biomarkers.

## Methods

### Data source and processing

Several related gene expression datasets were obtained from the GEO datasets (http://www.ncbi.nlm.nih.gov/geo/) with ‘metabolic syndrome’ and ‘coronary artery disease’ as keywords. The detailed information of datasets is presented in Supplementary materials Table 1 (see section on Supplementary materials given at the end of the article). This study employed a publicly accessible dataset that had previously received ethical approval.

Signal intensities from Agilent arrays were normalized by scaling to a trimmed mean of 100 and subsequently log_2_-transformed. Data quality was assessed using standard array quality control metrics, including the percentage of present probes, pairwise correlations, and overall signal intensity.

### DEG analysis

After raw data were preliminarily processed, the LIMMA package in R software 4.4.1 was utilized to compare gene expression of different groups to discern DEGs. The thresholds were *P*-value < 0.05 and fold change (FC) > 1.5. The Venn diagram was adopted to get shared DEGs between MetS and CAD.

### Weighted gene co-expression network analysis (WGCNA)

WGCNA was employed to construct co-expression gene networks, pinpoint functional modules, unveil the connection between gene networks and phenotypes, and identify core genes in the network. The co-expression network was built utilizing the WGCNA package, and the top 25% of genes with variance were then identified. Next, ineligible genes and samples were ruled out via the goodSamplesGenes function, and a scale-free co-expression network was mapped. An appropriate soft threshold power (*β*) was set to estimate intergenic adjacency, which was converted into a topological overlap matrix. Gene proportions and phase dissimilarities were examined. In addition, hierarchical clustering was conducted to detect modules using dynamic tree-cut functions. At last, correlations of gene significance with module membership were assessed, and module gene information was recorded.

### Identification of co-expressed genes through three machine learning (ML) algorithms

Disease occurrence served as the categorical variable. Least absolute shrinkage and selection operator (LASSO), support vector machine (SVM), and random forest (RF) were leveraged to investigate co-expressed genes between MetS and CAD.

First, the LASSO model was implemented within a nested tenfold cross-validation framework using the glmnet package to reduce the risk of over-fitting. Binomial was set, and the best lambda value was selected by lambda.min in the family parameter. Next, support vector machine-recursive feature elimination, SVM-RFE, was used to identify candidate hub genes. Using the e1071 and msvmRFE R packages, features were recursively eliminated in a backward manner according to their ranking scores. The SVM model was implemented within a nested cross-validation framework, with an outer tenfold loop for unbiased performance evaluation and an inner fivefold loop for SVM-RFE feature selection within each training set. During SVM-RFE, a linear kernel was used for feature ranking and recursive feature elimination. After feature selection, the final SVM classifier was constructed and evaluated using a radial basis function (RBF) kernel. This design ensured that feature selection was performed only on the training data and that held-out test samples were used exclusively for final prediction, thereby minimizing the risk of data leakage. Finally, RF analysis was performed using the randomForest R package to further screen significant candidate genes. The RF model was constructed with ntree = 500, and gene importance was evaluated based on the mean decrease in Gini index. Genes were ranked according to their importance scores, and the top-ranked genes were selected as significant candidate genes for subsequent analysis.

### Diagnostic value of identified hub biomarkers

The pROC package was adopted to assess the diagnostic sensitivity and specificity of hub genes. Through LASSO analysis, an integrated diagnostic model was established based on nine hub genes. The external dataset was utilized to corroborate the diagnostic value of hub genes and the model.

### Immune infiltration analysis

Twenty-eight immune cell infiltrations between the two diseases and normal controls were investigated through ssGSEA. The gene set variation analysis (GSVA) algorithm was applied. Spearman correlation analysis was made to determine the link among immune cells.

### Single-cell transcriptome data

Due to the absence of MetS sequencing data, we only downloaded the GSE159677 dataset for CAD, which comprised calcified atherosclerotic plaques and patient-matched proximal adjacent carotid arteries from 3 patients after carotid endarterectomy. Single-cell transcriptome data were preprocessed and analyzed using the Seurat R package, based on standard workflows described in the recent single-cell RNA-seq literature (11). Quality control was conducted by filtering cells with 200 < nFeature_RNA < 6,000 and percent.mt < 10. Then 2,000 highly variable genes were found utilizing the FindVariableFeatures function and normalized with the ScaleData function to lessen the impact of technical noise. Afterward, dimensionality reduction was implemented in the RunPCA function, and the first 20 principal components were identified. For batch-effect correction in multi-sample data, the Harmony function was used for data integration. Based on principal component analysis, Harmony iteratively removes systematic biases across datasets, thereby effectively integrating cells from different samples. After that, the FindNeighbours function was adopted to compute the distances between cells and plot a shared nearest neighbor graph. Subsequently, cell clustering was conducted with the FindClusters function in the Louvain algorithm, and a resolution parameter of 0.3 was set to determine cell subpopulations. UMAP dimensionality reduction was implemented in the RunUMAP function to visualize the integrated data. Finally, the automated annotations made by the SingleR software were merged with known cell marker genes and refined via manual corrections. Subsequently, the CellChat R package and the CellChatDB.human database were employed to explore the intercellular communication of different cell subpopulations for ligand–receptor interactions. Signaling pathways and receptor–ligand interactions were evaluated to reveal coordinated signaling across cell types.

### Peripheral blood samples and RT-qPCR

RT-qPCR was conducted as an exploratory validation in peripheral blood (*n* = 5). Total RNA was extracted via the MolPure® Blood RNA Kit (YEASEN, 19241ES50, China) and reverse transcribed to cDNA using the PrimeScript RT Reagent Kit. RT-qPCR was conducted in a total volume of 20 μL, including 4 μL of cDNA, 0.4 μL of specific primers, and 10 μL of SYBR Green PCR Master Mix (YEASEN, 11201ES).

Primer sequences were listed as follows:

*ADRB2*, forward sequence (5′–3′): TTG​CTG​GCA​CCC​AAT​AGA​AGC; reverse sequence (3′–5′): CAG​ACG​CTC​GAA​CTT​GGC​A.

*KDM6A*, forward sequence (5′–3′): GGA​CAT​GCT​GTG​TCA​CAT​CCT; reverse sequence (3′–5′): CTC​CTG​TTG​GTC​TCA​TTT​GGT​G.

Peripheral blood samples were collected at the Department of Endocrinology of the First Affiliated Hospital of Anhui Medical University. Informed consent was acquired from all participants, and the research followed the principles delineated in the Declaration of Helsinki. Clinical experiments were ratified by the Ethics Committee (PJ 2024-06-67).

### Statistical analysis

R software (version 4.4.1) was leveraged for bioinformatics analysis. Continuous variables were delineated as mean ± standard deviation (SD) and analyzed via the Wilcoxon signed-rank test. Spearman’s rank correlation analysis was adopted to assess the link between hub genes and diseases. RT-qPCR results were analyzed by GraphPad 8.0, and *P* < 0.05 implied statistical significance.

RT-qPCR results were calculated using the ΔΔCt method with GAPDH as the housekeeping gene and analyzed by GraphPad 8.0, and *P* < 0.05 implied statistical significance.

## Results

### Identification of shared DEGs

**Figure 1:**
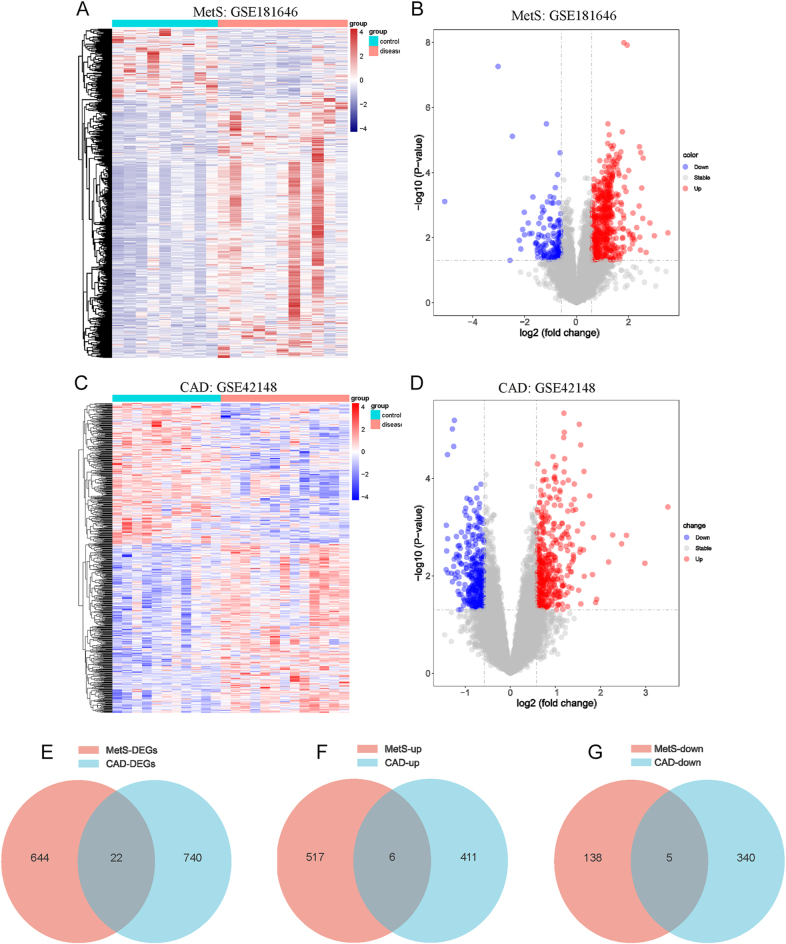
Identification of common DEGs between MetS and CAD. (A) The heatmap presents 644 DEGs in the MetS dataset (GSE181646). (B) The volcano plot shows the upregulated and downregulated DEGs in the MetS dataset (GSE181646). (C) The heatmap presents 740 DEGs in the CAD dataset (GSE42148). (D) The volcano plot shows the upregulated and downregulated DEGs in the CAD dataset (GSE42148). (E) The Venn diagram shows 22 overlapping DEGs from the MetS and CAD datasets. (F) The Venn diagram shows 6 overlapping upregulated DEGs between MetS and CAD datasets. (G) The Venn diagram shows 5 overlapping downregulated DEGs between MetS and CAD datasets. DEGs, differentially expressed genes; MetS, metabolic syndrome; CAD, coronary artery disease.

In total, 644 DEGs were found in the MetS dataset, with 523 upregulated and 143 downregulated genes in the heatmap (Fig. 1A and B). Meanwhile, 740 DEGs were found in the CAD dataset, containing 417 upregulated genes and 345 downregulated genes (Fig. 1C and D). At last, 22 overlapping DEGs were obtained (Fig. 1E; Table S2), including 6 upregulated and 5 downregulated shared genes (Fig. 1F and G; Table S3).

### Key modules screened by WGCNA

**Figure 2:**
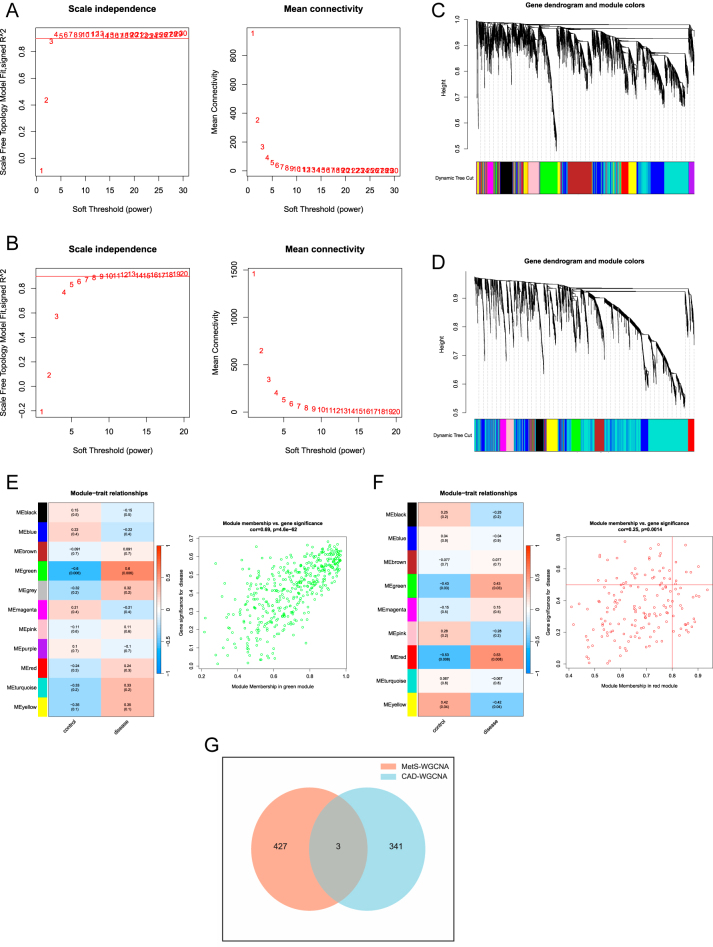
Screening for key modules by WGCNA. (A) Determination of soft-threshold power for MetS. (B) Calculation of soft-threshold power for CAD. (C) Cluster dendrogram of MetS highly connected genes in key modules. (D) Cluster dendrogram of CAD modules with highly connected genes. (E) Relationships between modules and traits in MetS. Correlations and *P*-values are included in each cell. (F) Module–trait relationships in CAD. A correlation and *P*-value are included in each cell. (G) The Venn diagram shows the common genes identified by WGCNA. MetS, metabolic syndrome; CAD, coronary artery disease.

WGCNA demonstrated that the best soft threshold was 6 in both the MetS and CAD datasets (Fig. 2A and B). 11 and 9 modules were determined in the MetS and CAD datasets, respectively (Fig. 2C and D). The green module showed positive correlations with MetS (*r* = 0.9), and the red module revealed significant associations with CAD (*r* = 0.53). Of note, a strong link was revealed between gene significance and module membership in the modules (correlation coefficient for MetS = 0.69 and correlation coefficient for CAD = 0.25), further suggesting the correlation of module genes with the diseases (Fig. 2E and F). Finally, 3 shared genes (*APOBEC3B*, *SGSM2,* and *LRRC32*) were screened, which may regulate MetS and CAD progression (Fig. 2G).

### Diagnostic genes identified through ML algorithms

**Figure 3:**
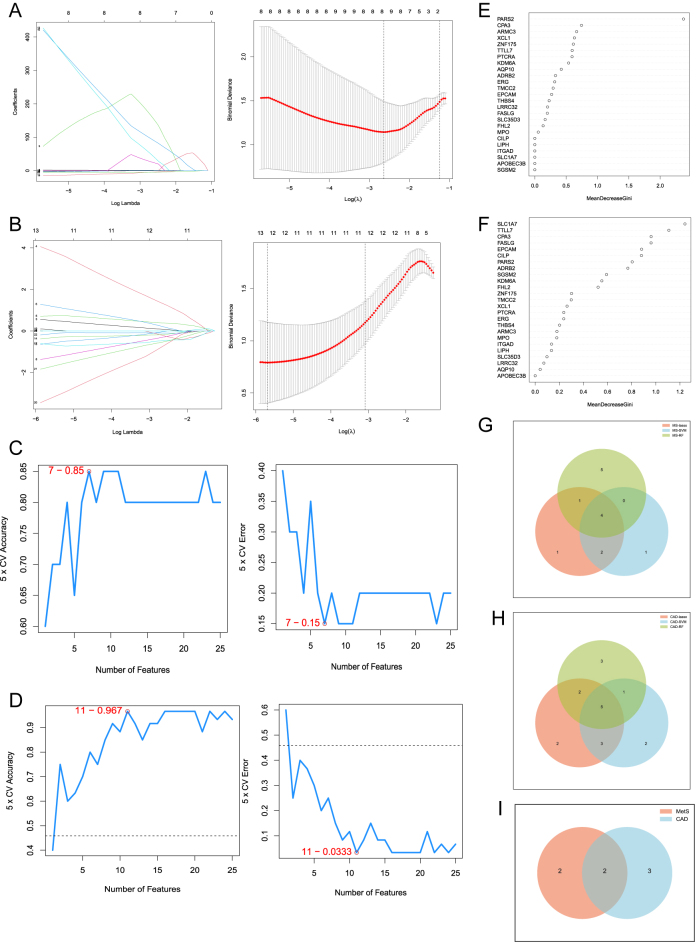
Identification of candidate MetS and CAD diagnostic genes using three machine learning algorithms. (A and B) Coefficient profile plots of the LASSO model for MetS (A) and CAD (B) show the final parameter selection λ (lambda). (C and D) Seven crosstalk genes were selected for MetS (C) and ten crosstalk genes are selected for CAD (D) by the SVM method, respectively. (E and F) MetS (E) and CAD (F) hub genes are ranked according to their discriminant ability in the RF algorithm. (G and H) The Venn diagram shows 4 candidate diagnostic genes in MetS (G) and 5 candidate diagnostic genes in CAD (H) by intersecting the results of three algorithms. (I) The Venn diagram shows 2 common diagnostic genes in MetS and CAD. LASSO, least absolute shrinkage and selection operator; SVM-RFE, support vector machine-recursive feature elimination; MetS, metabolic syndrome; CAD, coronary artery disease; RF, random forest.

The LASSO method pinpointed 9 genes in the MetS dataset and 12 genes in the CAD dataset (Fig. 3A and B). SVM pinpointed 7 genes with the lowest fivefold cross-validation (CV) and best 5-point CV error from the MetS dataset (Fig. 3C), and 11 genes from the CAD dataset (Fig. 3D) (Table S4). Immediately, WCGNA modules were integrated with DEGs, and the overlapping 25 hub genes were input into the RF classifier, and they were shown on the importance scale (Fig. 3E and F). Moreover, these three algorithms uncovered 4 overlapping biomarkers (*AQP10, PARS2, ADRB2,* and *KDM6A*) for the MetS group and 5 shared biomarkers (*CILP, CPA3, ADRB2, KDM6A,* and *SLC1A7*) for the CAD group (Fig. 3G and H). Finally, *ADRB2* and *KDM6A* were identified as candidate diagnostic biomarkers (Fig. 3I).

### Diagnostic value and validation of candidate biomarkers

**Figure 4:**
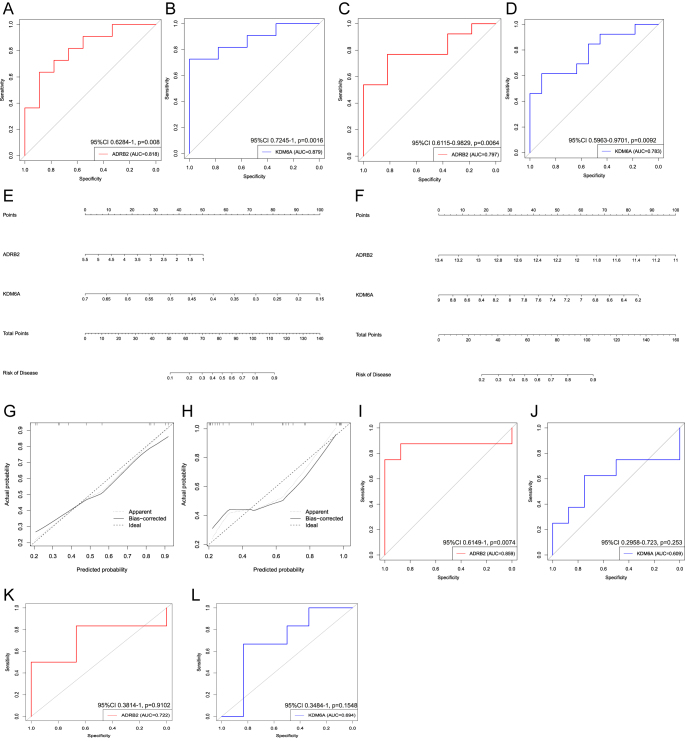
Selection and validation of the two common diagnostic genes. (A and B) ROC curve of *ADRB2* (A) and *KDM6A* (B) in the training group for MetS. (C and D) ROC curve of *ADRB2* (C) and *KDM6A* (D) in the training group for CAD. (E and F) Association between two common diagnostic genes and disease risk. (G and H) The calibration curves show the accuracy of model prediction for MetS (G) and CAD (H). (I and J) ROC curve of *ADRB2* (I) and *KDM6A* (J) in the validation set for MetS. (K and L) ROC curve of *ADRB2* (K) and *KDM6A* (L) in the validation set for CAD. MetS, metabolic syndrome; CAD, coronary artery disease; ROC, receiver operating characteristic.

AUC values of *ADRB2* (0.818) (95% *CI*: 0.6284–1, *P* = 0.008) and *KDM6A* (0.879) (95% *CI*: 0.7245–1, *P* = 0.0016) in the MetS dataset (Fig. 4A and B) and AUC values of *ADRB2* (0.797) (95% *CI*: 0.6115–0.9829, *P* = 0.0064) and *KDM6A* (0.783) (95% *CI*: 0.5963–0.9701, *P* = 0.0092) in the CAD dataset (Fig. 4C and D) were greater than 0.7, indicating good diagnostic performance and the potential as diagnostic markers for MetS and CAD. Furthermore, the expression levels of *ADRB2* and *KDM6A* were significantly downregulated in MetS and CAD groups compared to controls (Fig. 4E and F). Meanwhile, the calibration curve proved the model’s accuracy for the two diseases (Fig. 4G and H). In the validation set, different cohorts also exhibited favorable predictive efficacy, with AUCs of *ADRB*2 (0.859) (95% *CI*: 0.6149–1, *P* = 0.0074) and *KDM6A* (0.609) (95% *CI*: 0.2958–0.723, *P* = 0.253) in the MetS dataset (Fig. 4I and J) and AUCs of *ADRB2* (0.722) (95% *CI*: 0.3814–1, *P* = 0.9102) and *KDM6A* (0.694) (95% *CI*: 0.3484–1, *P* = 0.1548) in the CAD dataset (Fig. 4K and L).

### Immune cell infiltration and correlation analysis with shared hub genes

**Figure 5:**
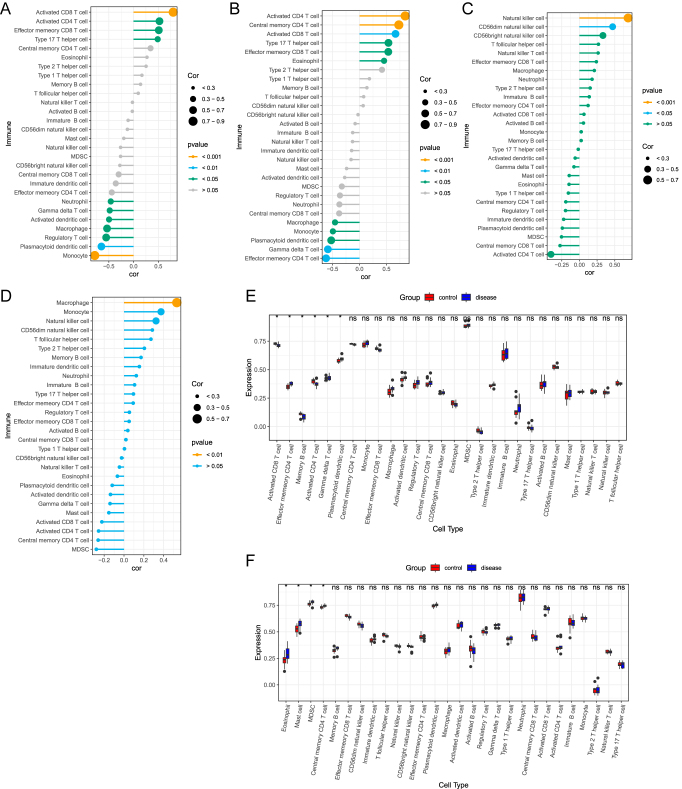
MetS and CAD immune cell composition. (A and B) Correlation between *ADRB2* (A) and *KDM6A* (B) expression and immune infiltration results in the MetS group. (C and D) Correlation between *ADRB2* (C) and *KDM6A* (D) expression and immune infiltration results in the CAD group. (E and F) The violin plots show that six immune cell types are significantly different between the MetS (E) and CAD (F) groups. MetS, metabolic syndrome; CAD, coronary artery disease; *P < 0.05.

A correlation was revealed between hub genes and immune infiltration in the MetS and CAD datasets. *ADRB2* was positively correlated with activated CD4^+^ T cells, activated CD8^+^ T cells, and effector memory CD8^+^ T cells, while negatively associated with monocytes, plasmacytoid dendritic cells, regulatory T cells, and macrophages. *KDM6A* possessed positive correlations with activated CD4^+^ T cells, central memory CD4^+^ T cells, activated CD8^+^ T cells, type 17 T helper cells, and effector memory CD8^+^ T cells, while negative links with gamma delta T cells, plasmacytoid dendritic cells, and effector memory CD4^+^ T cells in the MetS dataset (Fig. 5A and B). In the CAD dataset, *ADRB2* was positively linked with natural killer (NK) cells and CD56dim NK cells, while *KDM6A* was only positively correlated with macrophages (Fig. 5C and D). More notably, there were marked discrepancies in activated CD8^+^ T cells, effector memory CD4^+^ T cells, memory B cells, activated CD4^+^ T cells, and gamma delta T cells in the MetS dataset (Fig. 5E) and eosinophils, mast cells, MDSCs, and central memory CD4^+^ T cells in the CAD dataset (Fig. 5F) between the diseases and controls.

### Single-cell analysis of hub gene locations

**Figure 6:**
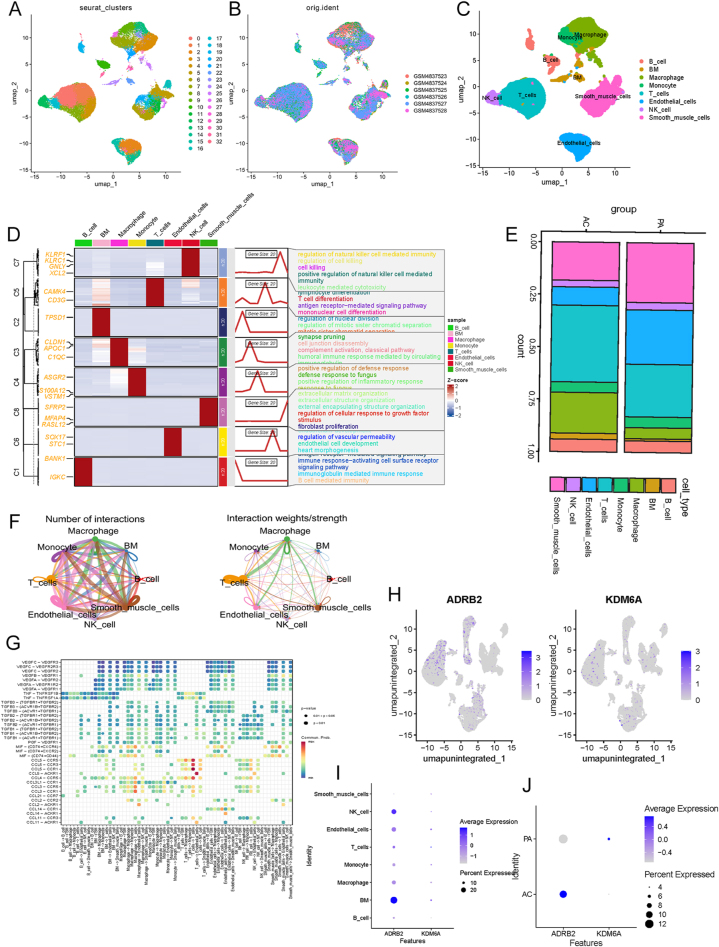
Single-cell analysis of hub gene expression profiles. (A) Uniform manifold approximation and projection (UMAP) visualization of all single cells after dimensionality reduction and unsupervised clustering. (B) The UMAP plot shows the distribution of cells from different original samples. (C) UMAP visualization of major annotated cell populations, including B cells, BM, macrophages, monocytes, T cells, endothelial cells, NK cells, and smooth muscle cells. (D) The heatmap shows expression patterns of representative marker genes across annotated cell populations, together with functional enrichment terms associated with each cell type or gene module. (E) The stacked bar plot shows relative proportions of major cell types in the AC and PA groups. (F) The cell–cell communication network analysis shows the number of interactions and interaction weights/strengths among the annotated cell populations. (G) The bubble plot shows selected ligand–receptor interactions among different cell populations. (H) The UMAP feature plots show the single-cell expression distribution of *ADRB2* and *KDM6A* across the integrated dataset. (I) The dot plot shows the expression levels of *ADRB2* and *KDM6A* across major annotated cell populations. (J) The dot plot compares expression patterns of *ADRB2* and *KDM6A* between the AC and PA groups. AC, atherosclerotic core; PA, proximal adjacent.

A total of 46,359 circulating cells were mapped to the cell atlas and annotated into eight major types, including B cells, BM, macrophages, monocytes, T cells, endothelial cells, NK cells, and smooth muscle cells. The expression distribution of several cell types was displayed in each sample and group by uniform manifold approximation and projection (UMAP) (Fig. 6A, B, C). Subsequently, gene ontology enrichment analysis discovered specific biological processes in each cell type (Fig. 6D). The cell atlas contained 6 tissue samples, and the cellular composition could be compared across samples. T cells and macrophages showed the most significant changes (Fig. 6E). To clarify the mechanisms of cell–cell interactions, we established separate intercellular communication networks using the CellChat method. The results indicated the most interactions between macrophages, monocytes, and other immune cells. Meanwhile, macrophages and T cells presented the strongest interaction (Fig. 6F). Several signaling pathways were primarily present, including the *CCL*, *MIF*, *TGFB*, and *VEGF*. The most robust interaction among T cells and macrophages was the CCL signal. *CCL5–CCR1/CCR5* strongly correlated (Fig. 6G). Finally, *ADRB2* expression was higher in several immune cells compared to *KDM6A* (Fig. 6H and I). Meanwhile, *ADRB2* and *KDM6A* were significantly expressed in proximal adjacent (PA) and atherosclerotic core (AC) groups (Fig. 6J).

### An exploratory validation of ADRB2 and KDM6A by RT-qPCR in human samples

**Figure 7:**
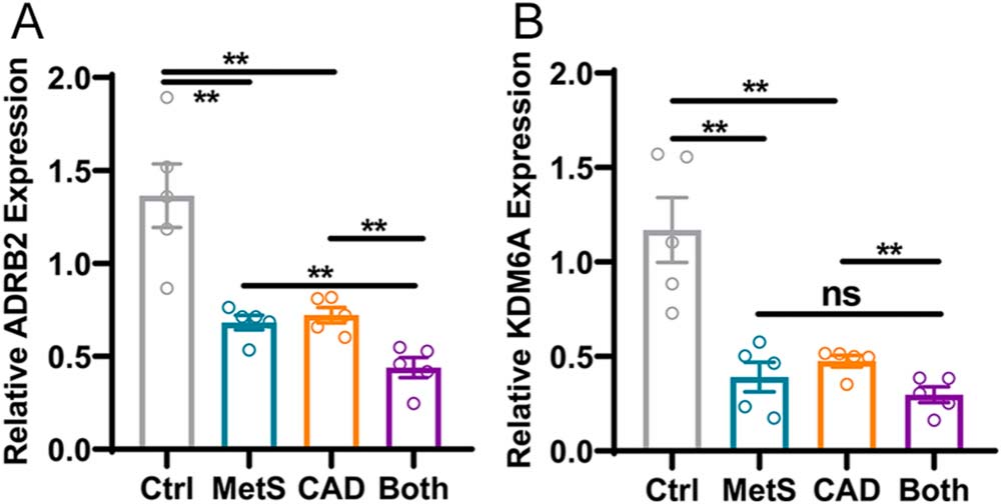
Validation of RT-qPCR in human samples. (A and B) Expression levels of *ADRB2* (A) and *KDM6A* (B) in MetS, CAD, and control groups (*n* = 5). Ctrl, control; MetS, metabolic syndrome; CAD, coronary artery disease; Both, group with MetS and CAD; ns, no significant; **P < 0.01.

RT-qPCR indicated that *ADRB2* and *KDM6A* were both downregulated in the peripheral blood of the MetS and CAD groups compared with the controls and lower in MetS with CAD individuals compared with the MetS or CAD groups (Fig. 7A and B).

## Discussion

At present, accumulating studies focus on the correlation between MetS and CVD. MetS and its components raise CVD risk and overall mortality (6, 12). Identification of shared biomarkers and pathogenesis is crucial for the diagnosis and treatment of the diseases. Herein, 4 microarray datasets from MetS and CAD were selected for various bioinformatic analyses, and the GEO database identified 2 shared DEGs (*ADRB2* and *KDM6A*) for MetS and CAD. Moreover, their diagnostic value was confirmed and validated by ROC curves, single-cell analysis, and RT-qPCR. These findings offer new clinical ideas and guidance for the diagnosis and management of MetS and CAD.

*ADRB2* was found to be under-expressed in MetS and CAD patients compared with normal controls and demonstrated favorable diagnostic values in MetS and CAD. MetS and its individual components mainly result from the complex interplay of genetic predisposition and environmental factors (13). The *ADRB2 gene* (encoding the β2-adrenergic receptor) has been implicated in human metabolic regulation, including modulation of adipose tissue thermogenesis, energy expenditure, and glucose homeostasis (14). Two population-based studies suggest that *ADRB2* genotypes and haplotypes may affect parameters related to MetS and insulin resistance in Korean adolescents, and genetic variations in common diseases are diverse (15, 16). In addition, *ADRB2* polymorphisms, such as rs1042714, have been shown to modulate changes in body composition, glucose metabolism, and insulin resistance following dietary intervention in adults (17). Notable associations of *ADRB2* polymorphisms with obesity, dyslipidemia, and DM have also been noted in Japanese (18). However, a case–control study of 7,808 white subjects failed to exhibit any consistent correlations of *ADRB2* polymorphisms with obesity, hypertension, or type 2 DM (19). These discrepancies may stem from variations in populations and polymorphisms of specific genes or ethnicities.

Previous studies elaborate that MetS and its components are closely linked with CVD risks (20). In this work, *ADRB2* expression may be negatively related to CAD. So far, published papers have explored the links between *ADRB2* polymorphisms and CVD risk and shown contradictory results. For instance, Wang *et al.* indicated that *ADRB2* rs1042713 and rs1042714 polymorphisms had an intimate link with CAD, and *ADRB2* polymorphisms might contribute to CVDs and serve as crucial markers for CAD diagnosis and prognosis (21, 22, 23). However, a follow-up study of 545 Han Chinese patients with CAD after percutaneous coronary intervention did not support an association between *ADRB2* polymorphisms (rs1042713 and rs1042714) and CVD risk. These inconsistent results are worth considering, and further studies are urgently needed to ascertain the correlation between *ADRB2* polymorphisms and CAD.

*KDM6A* is a histone demethylase that modulates the deletion of repressive trimethylation from histone H3 lysine 27, thus activating target genes (24). The *KDM6A* family (*KDM6A* and *KDM6B*) plays pivotal roles in cellular events, including differentiation, senescence, cancer, and inflammatory responses (25, 26, 27, 28). Recent literature focused on the critical roles of *KDM6A* in neoplastic diseases, such as bladder cancer, small-cell lung cancer, pancreatic cancer, multiple myeloma, and hepatocellular carcinoma (29, 30, 31, 32, 33). Our study showed reduced *KDM6A* levels in MetS and CAD cases compared to the control. Nevertheless, few articles have investigated the relationship between *KDM6A* and the two diseases. Chen et al. clarified *KDM6A* as an epigenetic switch that mediated macrophage polarization and disrupted energy balance, and macrophages with higher *KDM6A* considerably facilitated adipogenesis in white adipocytes and prevented thermogenesis in beige adipocytes (34). This positive effect is consistent with our results. Mechanically, *KDM6A* modulated Ire1α expression dependent on demethylase activity and stimulated M2 polarization of macrophages (34). It implies that *KDM6A* in macrophages stimulates obesity and MetS by repressing brown adipose tissue activity and white adipose tissue differentiation. Collectively, *KDM6A* may regulate MetS progression. In addition, Dai et al. stated that *KDM6A* regulated the proliferation and apoptosis of vascular smooth muscle cells in CAD (35). Further basic experiments and population-based studies are warranted to uncover the underlying mechanism and diagnostic value of *KDM6A* in MetS and CAD.

Our findings indicate that *ADRB2* and *KDM6A* expression are tightly linked to differential immune infiltration patterns in both MetS and CAD, suggesting distinct immunometabolic landscapes underlying these conditions. The positive associations of *ADRB2* with activated and effector memory T-cell subsets in the MetS dataset may indicate enhanced adaptive immune activation. This finding is consistent with accumulating evidence that obesity/MetS is characterized by chronic low-grade inflammation accompanied by T-cell infiltration in adipose tissue, sustained production of pro-inflammatory cytokines, and adipose tissue inflammation/remodeling that contributes to metabolic dysfunction (36, 37, 38). MetS is increasingly recognized as an immunometabolic disorder driven by coordinated alterations in innate and adaptive immune compartments. In particular, macrophage activation and dendritic cell-mediated immune modulation can amplify inflammatory signaling, thereby contributing to adipose tissue inflammation/remodeling and metabolic dysregulation (39, 40, 41, 42).

Moreover, the association of *KDM6A* with multiple T-cell subsets in the MetS dataset supports the notion that epigenetic regulators (including the *H3K27* demethylase *UTX/KDM6A*) can modulate T-cell programs and thereby shape immune landscapes in metabolic disorders (43). In contrast, in CAD, the correlations between *ADRB2* expression and NK cell subsets highlight the potential involvement of innate cytotoxic cells in atherosclerosis, aligning with recent evidence that natural killer cells and broader immune cell dynamics contribute to the pathogenesis of coronary artery disease and immune recruitment to vascular lesions (44). The observed discrepancies in specific T cell, B cell, NK cell, and myeloid populations between the disease and control groups further indicate that alterations in immune cell composition are disease-specific and may contribute to distinct mechanisms of inflammation and tissue damage in MetS versus CAD. Similarly, bioinformatic analyses have demonstrated differential infiltration of activated T cell subsets and NK cells in CAD patients compared with controls (45), while comprehensive reviews highlight the distinct roles of innate and adaptive immune cells, including macrophages, dendritic cells, T and B lymphocytes, in the pathogenesis of MetS and related cardiometabolic disorders (39). Moreover, comparative studies of coronary disease subtypes reveal divergent immune signatures across multiple immune lineages. These results support that MetS and cardiovascular diseases are not only metabolic in nature but are also characterized by profound immunological remodeling.

Moreover, the results of single-cell transcriptomic analysis revealed different abundance and interactions between immune cells across samples, with notable changes observed in T cells and macrophages. Recent single-cell studies have highlighted significant heterogeneity in immunity in cardiovascular diseases and MetS. In particular, macrophages, T cells, B cells, and NK cells exhibit different gene expression levels, which are correlated with disease progression and inflammatory signaling (46, 47). For instance, single-cell profiling in atherosclerosis has revealed that various immune cell subsets, including macrophage subpopulations and lymphocytes, contribute to lesion formation and inflammatory responses in vascular tissues (48). In addition, intercellular communication analyses in disease states have identified key signaling pathways, such as chemokine (e.g. *CCL*) and cytokine networks, which facilitate crosstalk between immune cells (49). This finding is consistent with our observation of prominent interactions between macrophages and T cells and robust *CCL5–CCR1/CCR5* signaling. The enrichment of pathways such as *MIF*, *TGFB*, and *VEGF* further emphasizes the complexity of immune modulation and cell–cell communication in disease pathophysiology. Importantly, the differential expression of hub genes, such as *ADRB2* and *KDM6A*, within specific immune subsets suggests that these genes may play regulatory roles in promoting immunometabolic reprogramming (27, 50). This aligns with emerging evidence that immune landscape remodeling influences disease mechanisms in coronary artery disease and MetS. Overall, our findings suggest that dynamic immune cell composition and interaction networks are crucial in understanding inflammatory mechanisms of specific diseases, which could inform targeted immunomodulatory strategies in cardiometabolic diseases.

Several limitations should be noted. First, this study relied on publicly available datasets, which inherently limited our ability to conduct experiments to explore the in-depth mechanism. Although significant associations were identified, the underlying biological mechanisms could not be fully elucidated in this study. Therefore, experimental validation and functional studies are needed to clarify the causal relationships and molecular mechanisms. Second, the sample size was relatively limited, which may reduce the statistical power and limit the generalizability of the findings. Although the datasets were obtained from a publicly available database with standardized data collection and quality control procedures, the number of eligible samples was small, and the sample size could not be further expanded. Therefore, the results should be interpreted with caution, and future studies with larger and independent cohorts are warranted to validate and extend our findings. Third, little is known about how raw data were processed, and the datasets used were analyzed by microarray, lagging behind advanced sequencing technologies. The inherent characteristics of public datasets challenge the accuracy of our analysis. Finally, the RT-qPCR experiment was performed as an exploratory validation in a small set of peripheral blood specimens (*n* = 5), which may increase uncertainty in estimating the effect size. Nevertheless, the concordant results across multiple independent transcriptomic datasets, together with the convergence of three feature-selection methods, support the robustness of the identified two-gene signature. Further large-scale experimental validation is warranted.

## Conclusion

We evaluated transcriptome data from MetS and CAD patients, identified shared DEGs and hub genes, and performed WGCNA, ML algorithms, single-cell analysis, and RT-qPCR. Our results suggest that *ADRB2* and *KDM6A* may serve as potential biomarkers for MetS and CAD. This study will aid in exploring the molecular mechanisms underlying MetS and CAD.

Overall, this study highlights a potential approach for the evaluation of MetS and CAD risk. Genetic testing might, in the future, help estimate risk and assist in early intervention, but these findings remain preliminary and require further validation in larger, independent cohorts and clinical studies.

## Supplementary materials



## Declaration of interest

The authors declare that there is no conflict of interest that could be perceived as prejudicing the impartiality of the work reported.

## Funding

This work was supported by the National Science Fund for Distinguished Young Scholars (82504438), the Key Project of the Anhui Provincial Health Research Program (AHWJ2023A10010), the Anhui Provincial Special Program for Clinical Research Translation (202427b10020078), and the Science and Technology Project in Henan (242102311009).

## Author contribution statement

NW and KH contributed to methodology, software, investigation, resources, and writing of the original draft. SZ and HZ contributed to data curation, validation, and formal analysis. XL, YZ, TW, and TP contributed to supervision and writing, reviewing, and editing. YZ, KH, and TP contributed to project administration and funding acquisition. All authors read and approved the final manuscript.

## Ethics approval

This paper was approved by the Ethics Committee of The First Affiliated Hospital of Anhui Medical University (Approval No. PJ2024-06-67) and was conducted in accordance with the Declaration of Helsinki.

## Consent to participate

Informed consent was obtained from all participants included in the study.

## Data availability

The datasets used in this study are all available from the GEO database (http://www.ncbi.nlm.nih.gov/geo/), and other data are available from the corresponding authors upon reasonable request.
