# Supplementary material for: Shared diagnostic biomarkers in metabolic syndrome and coronary artery disease identified by integrated bioinformatics and machine learning
Source: Endocr Connect. 2026 Jun 8;15(6):e260240. doi: 10.1530/EC-26-0240 (PMC13250652; doi:10.1530/EC-26-0240)
Supplement: Supplementary file 1 [file supplementary_materials.pdf]

Table S1. The detail information of several datasets used in this study.

Dataset	Disease	Sample type	Sample size	Platform	Role
GSE181646	MetS	RNA-seq	11 MetS and 9 controls	GPL30209	Train set
GSE145412	MetS	RNA-seq	8 MetS and 8 controls	GPL18573	Validation set
GSE42148	CAD	RNA-seq	13 CAD and 11 controls	GPL30209	Train set
GSE98583	CAD	RNA-seq	6 CAD and 6 controls	GPL571	Validation set
GSE159677	CAD	Single cell RNA-seq	3 CAD and 3 controls	GPL18573	Single-cell analysis

Table S2. Overlapping DEGs between MetS and CAD.

AQP10
ARMC3
CILP
CPA3
EPCAM
ERG
FHL2
LIPH
MPO
PARS2
PTCRA
SLC35D3
THBS4
TMCC2
TTLL7
ZNF175
ADRB2
FASLG
ITGAD
KDM6A
SLC1A7
XCL1

Table S3. Overlapping up- and down-regulated DEGs between MetS and CAD

Shared-up	Shared-down
THBS4	*FASLG*
MPO	*KDM6A*
CPA3	*ADRB2*
CILP	*XCL1*
EPCAM	*SLC1A7*
EPCAM	

Table S4. Identification of hub genes through machine learning algorithms.

LASSO		SVM	
MetS	CAD	MetS	CAD
AQP10	*CILP*	*PARS2*	*KDM6A*
EPCAM	*CPA3*	*AQP10*	*CPA3*
PARS2	*EPCAM*	*ADRB2*	*ERG*
THBS4	*ERG*	*ITGAD*	*SLC1A7*
TTLL7	*LIPH*	*KDM6A*	*CILP*
ADRB2	*TMCC2*	*EPCAM*	*THBS4*
ITGAD	*TTLL7*	*MPO*	*LIPH*
KDM6A	*ADRB2*		*PARS2*
LRRC32	*ITGAD*		*ITGAD*
	KDM6A		*ADRB2*
	SLC1A7		*ZNF175*
	XCL1		
